# Comparative analyses of two Geraniaceae transcriptomes using next-generation sequencing

**DOI:** 10.1186/1471-2229-13-228

**Published:** 2013-12-29

**Authors:** Jin Zhang, Tracey A Ruhlman, Jeffrey P Mower, Robert K Jansen

**Affiliations:** 1 Department of Integrative Biology and Institute of Cellular and Molecular Biology, The University of Texas at Austin, 205 W. 24th St. Stop C0930, Austin, TX 78712, USA; 2 Center for Plant Science Innovation and Department of Agronomy and Horticulture, University of Nebraska-Lincoln, Lincoln, NE 68588, USA; 3 Genomics and Biotechnology Section, Department of Biological Sciences, Faculty of Science, King Abdulaziz University, Jeddah 21589, Saudi Arabia

## Abstract

**Background:**

Organelle genomes of Geraniaceae exhibit several unusual evolutionary phenomena compared to other angiosperm families including accelerated nucleotide substitution rates, widespread gene loss, reduced RNA editing, and extensive genomic rearrangements. Since most organelle-encoded proteins function in multi-subunit complexes that also contain nuclear-encoded proteins, it is likely that the atypical organellar phenomena affect the evolution of nuclear genes encoding organellar proteins. To begin to unravel the complex co-evolutionary interplay between organellar and nuclear genomes in this family, we sequenced nuclear transcriptomes of two species, *Geranium maderense* and *Pelargonium* x *hortorum.*

**Results:**

Normalized cDNA libraries of *G. maderense* and *P.* x *hortorum* were used for transcriptome sequencing. Five assemblers (MIRA, Newbler, SOAPdenovo, SOAPdenovo-trans [SOAPtrans], Trinity) and two next-generation technologies (454 and Illumina) were compared to determine the optimal transcriptome sequencing approach. Trinity provided the highest quality assembly of Illumina data with the deepest transcriptome coverage. An analysis to determine the amount of sequencing needed for *de novo* assembly revealed diminishing returns of coverage and quality with data sets larger than sixty million Illumina paired end reads for both species. The *G. maderense* and *P*. x *hortorum* transcriptomes contained fewer transcripts encoding the PLS subclass of PPR proteins relative to other angiosperms, consistent with reduced mitochondrial RNA editing activity in Geraniaceae. In addition, transcripts for all six plastid targeted sigma factors were identified in both transcriptomes, suggesting that one of the highly divergent *rpoA*-like ORFs in the *P.* x *hortorum* plastid genome is functional.

**Conclusions:**

The findings support the use of the Illumina platform and assemblers optimized for transcriptome assembly, such as Trinity or SOAPtrans, to generate high-quality *de novo* transcriptomes with broad coverage. In addition, results indicated no major improvements in breadth of coverage with data sets larger than six billion nucleotides or when sampling RNA from four tissue types rather than from a single tissue. Finally, this work demonstrates the power of cross-compartmental genomic analyses to deepen our understanding of the correlated evolution of the nuclear, plastid, and mitochondrial genomes in plants.

## Background

Four remarkable evolutionary phenomena are associated with organellar genomes of Geraniaceae. First, mitochondrial genomes show multiple, major shifts in rates of synonymous substitutions, especially in the genus *Pelargonium*[1,2]. Rate fluctuations of such magnitude have been documented in only two other plant lineages, *Plantago*[3] and *Silene*[4-6]. Second, mitochondrial genomes have experienced extensive loss of genes and sites of RNA editing. At least 12 putative gene losses have been documented in *Erodium*[7], and mitochondrial genes sequenced from *Pelargonium* x *hortorum* had a drastic reduction in predicted or verified RNA editing sites compared to all other angiosperms examined [1]. Third, genome-wide comparisons of nucleotide substitutions in plastid DNA indicated rapid rate acceleration in genes encoding ribosomal proteins, RNA polymerase, and ATP synthase subunits in some lineages. In the case of RNA polymerase genes there was evidence for positive selection [8,9]. Fourth, plastid genomes of Geraniaceae are the most highly rearranged of any photosynthetic land plants examined [10-13]. Multiple and extreme contractions and expansions of the inverted repeat (IR) have resulted in genomes with both the largest IR (74,571 bp, [11]) as well as the complete loss of this feature [12,13]. Considerable accumulation of dispersed repeats associated with changes in gene order has been documented along with disruption of highly conserved operons and repeated losses and duplications of genes [12]. In *P.* x *hortorum* plastids, these genomic changes have generated several fragmented and highly divergent *rpoA*-like ORFs of questionable functionality [8,10-12], despite the fact that *rpoA* encodes an essential component of the plastid-encoded RNA polymerase (PEP).

Because nuclear genes supply both organelles with the majority of their proteins, it is likely that the extensive organellar genomic upheaval in Geraniaceae will also influence the evolution of organelle-targeted genes in the nuclear genome. For example, given the drastic reduction of RNA editing in Geraniaceae mitochondrial transcripts, it is reasonable to expect a correlated reduction of nucleus-encoded pentatricopeptide repeat (PPR) proteins, many of which are critical for organellar RNA editing [14-17]. The uncertain status of the *P.* x *hortorum* plastid-encoded *rpoA* gene is also likely to have nuclear consequences. If this plastid gene is not functional, then a functional copy might have been relocated to the nuclear genome, which has only occurred once in the evolution of land plants in mosses [18,19]. Alternatively, it is possible that PEP has become nonfunctional in *P.* x *hortorum*, as observed in the holoparasite *Phelipanche aegyptiaca*[20]. In *P. aegyptiaca*, loss of all plastid-encoded PEP components (*rpoA*, *rpoB*, *rpoC1* and *rpoC2*) resulted in the parallel loss of the requisite nucleus-encoded components (sigma factors) that assemble with the plastid encoded proteins to form the core of the PEP holoenzyme [20]. In contrast, if the highly divergent plastid *rpoA* gene is still functional in *P.* x *hortorum*, then the typical set of sigma factors should be present in the nuclear genome.

One prerequisite to begin to address the effects of organellar genomic upheaval on the nuclear genome in Geraniaceae, is availability of nuclear sequence information. Transcriptome sequencing provides a tractable proxy for nuclear gene space. The use of next-generation sequencing (NGS) for transcriptome sequencing is widespread because volumes of data can be generated rapidly at a low cost relative to traditional Sanger sequencing [21]. The assembly of reads into contigs may be executed using a *de novo* or a reference-based approach [22]. In studies of non-model organisms, *de novo* assembly is more commonly used due to the absence of a closely related reference [23,24]. A survey of recent transcriptome studies in comparative biology demonstrates that most sequencing projects are focusing on non-model organisms where little or no genomic data is available [22,25-31]. The lack of a reference genome makes the reconstruction and evaluation of the transcriptome assembly challenging. Several issues must be addressed when performing transcriptome sequencing of non-model organisms, including which NGS platform should be employed, how much sequence data is needed to provide a comprehensive transcriptome, which assembler should be utilized, and what tissues should be sampled.

This paper provides a comprehensive comparison of the transcriptomes of two non-model plant species, *Pelargonium* x *hortorum* and *Geranium maderense*, from the two largest genera of Geraniaceae. There were three primary goals for the initial comparative transcriptome analysis in Geraniaceae: (1) What are the best sequencing platforms and assembly methods for generating a high-quality transcriptome that broadly covers gene space in the absence of a reference genome? (2) Does sequencing from multiple tissue types improve the breadth of transcriptome coverage? (3) Are there any losses of PPR proteins involved in RNA editing and sigma factors associated with PEP in Geraniaceae?

## Results

### Ribosomal RNA content and Illumina library complexity

To assess the efficiency of ribosomal RNA (rRNA) depletion in Geraniaceae transcriptome libraries rRNA contigs were identified using rRNA from *Arabidopsis thaliana* as a reference. All Illumina reads (146,690,142 reads for *Geranium maderense* and 148,749,374 reads for *Pelargonium* x *hortorum*) were mapped to rRNA contigs as described in methods, and 0.7% and 2% of the reads of *G. maderense* and *P.* x *hortorum* were identified as rRNA reads, respectively. Library complexity was analyzed using Picard [32] and rRNA reads were eliminated prior to the analysis. The percentages of unique start sites were 42.7% and 46.1% for *G. maderense* and *P*. x *hortorum*, respectively. The values for rRNA content and library complexity were comparable to other transcriptome analyses using similar approaches [33,34].

### Assessment of sequencing platforms and assemblers for transcriptome assembly

**Figure 1:**
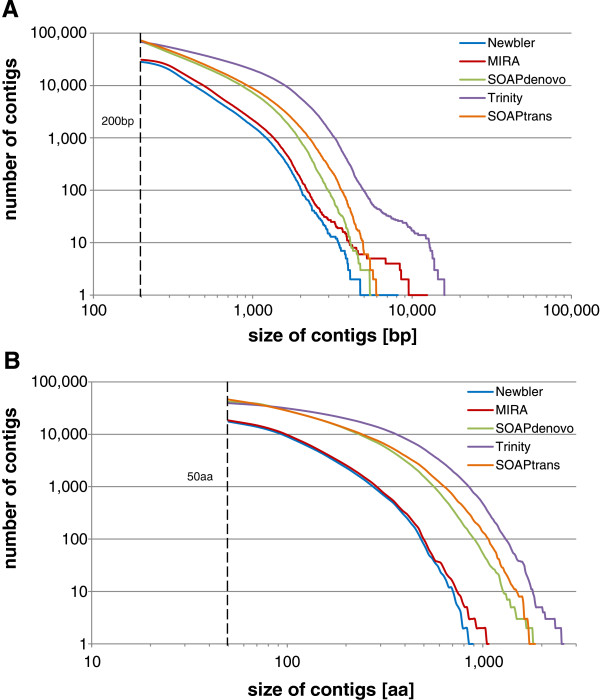
**Contig length. (A)** Contig length distribution. The vertical dashed line shows the arbitrary cutoff of 200 base pairs (bp). Contigs shorter than 200 bp were disregarded for this analysis. **(B)** Translated contigs length distribution. The vertical dashed line shows the arbitrary cutoff of 50 amino acids (aa). Contigs shorter than 50 aa were disregarded for this analysis.

To determine the optimal sequencing and assembly strategy, the efficacy of five different assemblers was examined using two initial data sets generated by Roche/454 FLX and Illumina Hiseq 2000 platforms for *P.* x *hortorum*. The Illumina run produced approximately 40 times more sequence data than the 454 run, even though the cost of the 454 data was at least four times more than the Illumina data (Table 1). A comparison of basic assembly statistics (Table 2) showed that the Trinity assembler outperformed all other platform/software combinations in terms of number of contigs, number of assembled nucleotides, mean and maximum contig length, and N50. More generally, the Illumina assemblers consistently outperformed the 454 assemblers, although the MIRA and Newbler 454 assemblers produced longer maximal contigs than SOAPdenovo and SOAPdenovo-trans (SOAPtrans). To determine the amount of usable protein sequence information generated by each assembler, the assemblies were translated as described in methods and compared (Table 3). Again, the Illumina assemblers outperformed the 454 assemblers in all metrics, with the Trinity assembler providing the most amino acids with the longest mean and maximal sequences. The length distribution of assembled nucleotides and translated amino acids further confirms that Trinity outperformed SOAPdenovo and SOAPtrans, and all three Illumina assemblers outperformed the 454 assemblers (Figure 1).

**Table 1:** **The *Pelargonium *** **x *hortorum *** **transcriptome dataset read statistics**

**Technology**	**Number of trimmed reads**	**Number of trimmed bases**	**Max read length**	**Min read length**
**454**	472,268	119,394,317	828	50
**Illumina**	46,475,742	4,674,574,200	100	100

**Table 2:** **Basic assembly statistics for the *Pelargonium *** **x *hortorum *** **transcriptome**

	**Newbler**	**MIRA**	**SOAPdenovo**	**Trinity**	**SOAPtrans**
**Number of nonredundant contigs**	28,182	30,947	67,028	67,614	62,470
**Total bases**	12,972,883	15,326,277	39,088,184	58,210,111	33,057,051
**Max contig length**	8,147	12,431	6,616	16,017	7,574
**Mean contig length**	460	495	583	860	529
**N50**	478	525	782	1,319	678

**Table 3:** **Translated contig statistics for *Pelargonium *** **x *hortorum***

	**Newbler**	**MIRA**	**SOAPdenovo**	**Trinity**	**SOAPtrans**
**Number of translated contigs**	18,525	19,279	42,907	39,742	44,379
**Total amino acids (AA)**	2,413,770	2,575,430	8,363,275	11,058,408	7,697,127
**Max translated AA length**	902	1,086	1,902	2,618	2,520
**Mean translated AA length**	130	133	195	278	173
**N50**	145	145	278	387	230

**Figure 2:**
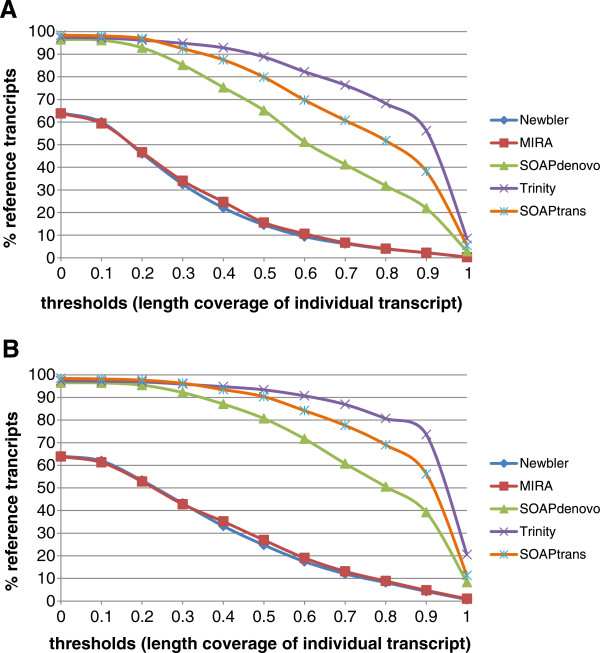
**Contiguity (A) and completeness (B) of different assemblers at different thresholds.** The assemblies were aligned with two published reference data bases: 357 ultra-conserved ortholog (UCO) coding sequence [35] and 959 single copy nuclear genes [36].

**Figure 3:**
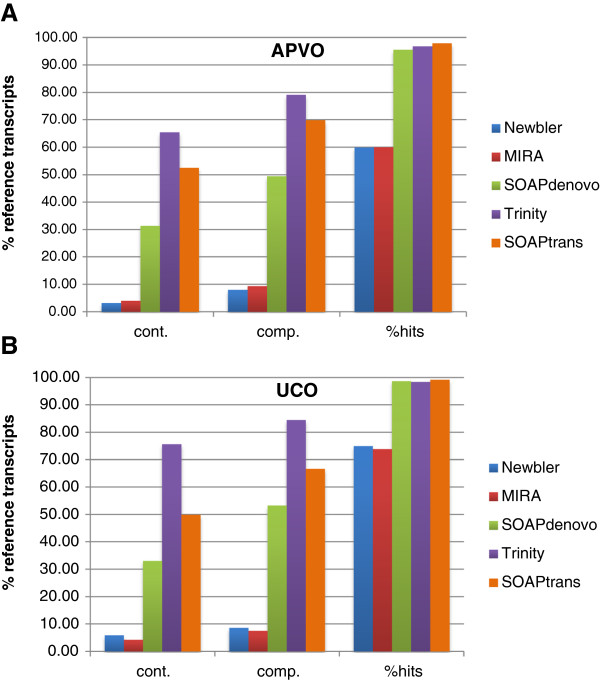
**Completeness and contiguity results at threshold 80% using two published reference protein sets.** Data sets: **(A)** 959 single copy nuclear genes (APVO); **(B)** 357 ultra-conserved ortholog (UCO) coding sequence [35,36]. Cont = contiguity, comp = completeness, % hits = percentage of hits in reference transcriptome.

Two important considerations in assembly analysis are the breadth of gene space coverage and the degree of coverage fragmentation. A good assembler should generate high-quality assemblies that contain as many reference transcripts as possible, and each reference transcript should be covered as completely as possible with a single long contig rather than a combination of several short contigs. To assess assembly coverage and fragmentation, two published data bases were used, 357 ultra-conserved ortholog (UCO) coding sequence [35] from *Arabidopsis* and 959 single copy nuclear genes shared between *Arabidopsis*, *Oryza*, *Populus*, and *Vitis*[36]. Trinity and SOAPtrans outperformed all other assemblers in terms of the percentage of reference genes identified, completeness of coverage (i.e. fraction of reference gene coverage by one or more contigs), and contiguity of coverage (i.e. fraction of reference gene coverage by a single long contig), with Trinity performance slightly better than SOAPtrans at higher thresholds (Figures 2 and 3).

To examine whether the superior performance of Trinity and SOAPtrans was due to the much larger amount (40 times) of Illumina data than 454 data, the Illumina assemblers were re-anlayzed using a data set containing 1/40^th^ of the Illumina reads (Additional file 1). In terms of contiguity and completeness, the performance of Trinity using the reduced Illumina data set remained superior to the 454 programs (Newbler, MIRA) that used the entire 454 data sets. In contrast, the performance of SOAPdenovo and SOAPtrans were noticeably worse with the reduced Illumina data set than with the full data set, producing results that were generally worse than the original 454 assemblies.

### Effect of sequencing depth on assembly coverage breadth and fragmentation

**Figure 4:**
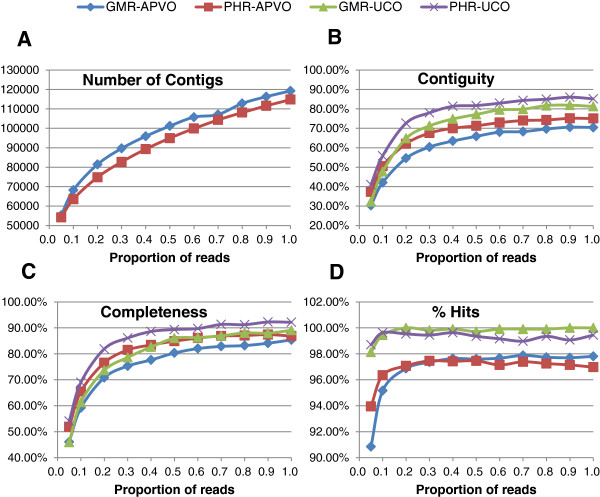
**Comparisons of *Geranium maderense *and *Pelargonium *x *hortorum *for four assembly parameters using different percentages of sequencing reads. (A)** number of contigs, **(B)** contiguity, **(C)** completeness, and **(D)** percentage of hits. For completeness and contiguity two published reference protein sets were used (357 ultra-conserved ortholog (UCO) coding sequence [35] and 959 single copy nuclear genes [36]). Assemblies were aligned with the reference data sets using BLASTX with an E-value of 1 E-10.

To determine how much sequence data is needed to assemble a high-quality transcriptome with broad coverage, 146,690,142 reads for *G. maderense* and 148,749,374 reads for *P.* x *hortorum* were generated on the Illumina Hiseq 2000 platform assembled using Trinity with different increments of reads from 5% to 100% of the total. While the number of contigs assembled continued to increase with increasing numbers of reads (Figure 4A), the percentage of reference genes recovered and their contiguity and completeness plateaued at approximately 40% of the total reads (Figure 4B-D). Including the remaining 60% of the reads increased contiguity and completeness by only 1% to 2% (Figure 4B-C). Although there were more translated contigs of *G. maderense* than *P.* x *hortorum*, the contiguity and completeness of both species were very similar.

**Figure 5:**
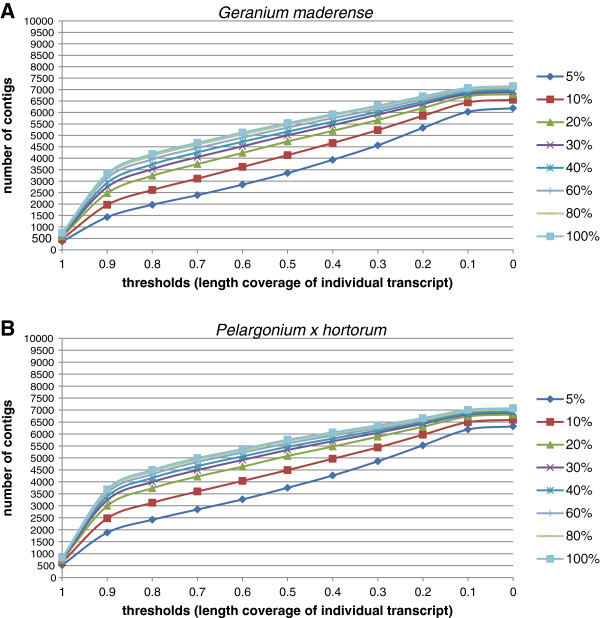
**Contiguity of *Geranium maderense *(A) and *Pelargonium x hortorum *(B) at different threshold values with differ percentages of reads using all *Arabidopsis *proteins from Uniprot/Swissprot database [**[85]**].** Assemblies were aligned with the database using BLASTX with an evalue of 1 E-10.

Although increasing the number of reads beyond 10% contributed little to finding novel hits to the local *Arabidopsis* data base, increasing the amount of data did help extend the existing contigs and generate longer alignments to reference genes. To evaluate this, the contiguity of all contigs relative to the two published databases was calculated at different contiguity thresholds up to 100% (Figure 5). The inclusion of more reads generated assemblies with higher contiguity, especially when contiguity thresholds were greater than 50%. To allow for the high level of sequence divergence between Geraniaceae and *Arabidopsis*, the number of contigs that had contiguity thresholds ≥80% was calculated. When 100% of the reads were used 4185 contigs and 4494 contigs were found in *G. maderense* and *P.* x *hortorum*, respectively. Reducing the read input to 40% reduced contiguity values by 7% (4163/4494) in *G. maderense* and 11% (3731/4185) in *P.* x *hortorum*.

### Functional assessment of Geraniaceae nuclear transcriptomes

**Figure 6:**
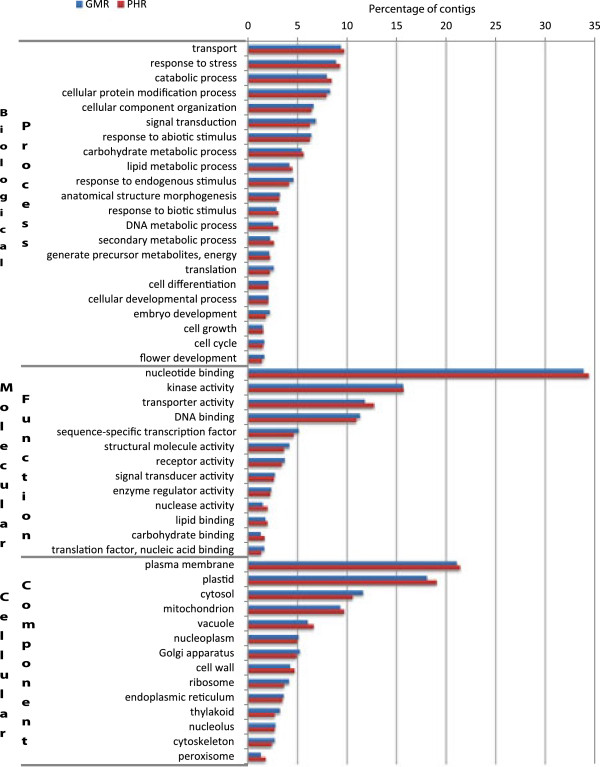
**Gene ontology assignments for *Geranium maderense *(GMR) and *Pelargonium *x *hortorum*.** The proportion of annotated contigs in all categories with >1% representation within the ontology (GO) categories for cellular component, molecular function, and biological process.

The assemblies generated using 100% of the reads for both Geraniaceae species were used for functional annotation. Assemblies were first aligned against the NCBI nr database and the alignment results were used to generate the gene ontology (GO) terms. Of the 114,762 contigs in *P.* x *hortorum*, 56,283 (49%) had blast hits; 42,506 (37%) were annotated and 222,765 GO terms were retrieved (Table 4). Of the 119,217 contigs in *G. maderense*, 76,332 (64%) had blast hits; 58,461 (49%) were annotated (Table 4) and 311,108 GO terms were retrieved. The annotation files are shown in Additional file 2. The distribution of gene ontology annotations was examined using GO-slim (plant) ontology to compare the transcriptomes of *G. maderense* and *P*. x *hortorum*. Although the number of annotated contigs differed substantially between the two transcriptomes (Table 4), the proportion of annotated contigs in all categories with >1% representation within the categories cellular component, molecular function, and biological process were very similar (Figure 6). This similarity persists even though only emergent leaves were sampled for *G. maderense* versus four tissue types (emergent and expanded leaves, roots and flowers) for *P.* x *hortorum*.

**Table 4:** **Statistics of transcriptome annotations for *Geranium maderense *** **(GMR) and *Pelargonium *** **x *hortorum *** **(PHR)**

	**GMR**	**PHR**
Total contigs	119,217	114,762
Aligned contigs	76,332	56,283
Annotated contigs	58,461	42,506
Assigned GO terms	311,108	222,765
Assigned EC	25,533	19,354
Contigs with EC	20,337	15,252

GO = Gene Ontology; EC = Enzyme Code.

To more directly address the question whether sequencing from multiple tissue types improves the breadth of transcriptome coverage, orthologous genes between *G. maderense* and *A. thaliana* and between *P.* x *hortorum* and *A. thaliana* were identified. Of the 35,386 protein sequences from *A. thaliana*, the *G. maderense* assembly had homologs to 11,131 sequences and the *P.* x *hortorum* assembly had homologs to 11,583 sequences. The comparable numbers of orthologous genes found for the two Geraniaceae species indicated that there was little improvement on the breadth of transcriptome coverage by sequencing from multiple tissue types (1 versus 4 tissues for *G. maderense* and *P*. x *hortorum*, respectively).

### Identification of selected organelle targeted genes

Pentatricopeptide repeat proteins (PPRs) are a large family of RNA binding proteins encoded by over 400 genes in angiosperms; most are organelle targeted and involved in regulating organelle gene expression. The transcriptomes of *P.* x *hortorum* and *G. maderense* were annotated using 429 *Arabidopsis* PPR sequences as a reference database (Table 5). The overall number of PPR genes varied considerably between the two Geraniaceae and *Arabidopsis*, with PPR gene number reduced in *P.* x *hortorum*. The numbers of P class PPR genes were found to be similar in all three species, whereas many fewer PLS class genes were found in the Geraniaceae, especially in *P.* x *hortorum*.

**Table 5:** **PPR protein**^**a **^**and sigma factor**^**b **^**distribution**

	***Arabidopsis thaliana***	***Geranium maderense***	***Pelargonium *** **x *hortorum***
PPR proteins	429	523	315
P class	238	387	262
PLS-E class	105	96	22
PLS-DYW class	86	40	31
Sigma factors	6	10	6
Sig 1	1	1	1
Sig 2	1	4	1
Sig 3	1	1	1
Sig 4	1	1	1
Sig 5	1	3	1
Sig 6	1	1	1

^a^PPR protein data of *Arabidopsis* are from Small and Peeters [92]. The PPR class represents the number contigs longer than 150 aa, which is the minimum length of PPR proteins identified in *Arabidopsis*. ^b^The number of total contigs and the number of intact contigs aligned to the reference sigma factors are shown. Intact contigs are those with start/stop codons on 5′ and 3′ ends, and without any internal stop codons.

Sigma factors are nuclear encoded, plastid targeted proteins that assemble with four plastid encoded proteins (*rpoA*, *rpoB*, *rpoC1* and *rpoC2*) to form the core of the PEP holoenzyme. At least one copy of each of the six *Arabidopsis* sigma factors was detected in both the *G. maderense* and *P. x hortorum* transcriptomes (Table 5). The nucleotide and amino acid sequence identities between *Arabidopsis*/*Geranium* and *Arabidopsis*/*Pelargonium* for all six sigma factors were very similar (Table 6). The four contigs from *G. maderense* that aligned to sigma factor 2 were similar to each other in nucleotide sequence identity (87%), suggesting that they may represent variant copies of the same gene. Two of the three contigs from *G. maderense* that aligned to sigma factor 5 were very similar to each other but less so to the third contig (98% versus 71% nucleotide sequence identity). Sigma factors 2 and 6 were each represented by two *P.* x *hortorum* contigs, however only one of the contigs for each sigma factor appeared functional having start/stop codons at the 5′ and 3′ ends and lacking internal stop codons. Further experiments are needed to determine if the copies with internal stop codons are pseudogenes or assembly artifacts.

**Table 6:** **Sequence identities between intact contigs in Geraniaceae and *Arabidopsis thaliana *** **sigma factors**

***Arabidopsis thaliana***	***Geranium maderense***		***Pelargonium *** **x *hortorum***	
**Sequence identity (%)**^**a**^	**Nucleotide**	**Amino acid**	**Nucleotide**	**Amino acid**
Sig 1	64.5	52.4	61.1	50.4
Sig 2	62.6	48.9	62.5	47.1
Sig 3	57.7	39.9	58.6	43.3
Sig 4	58.6	42.6	58.3	43.5
Sig 5	64.3	54.1	65.8	55.0
Sig 6	58.6	41.1	59.6	42.4

^a^In cases where there is more than one intact contig for a sigma factor, the one with highest sequence identity to *Arabidopsis* was selected for comparison.

## Discussion

### Strategies for *de novo* assembly of transcriptomes

The use of NGS platforms is widespread and is applied in many research fields as volumes of data can be generated rapidly at a low cost relative to traditional Sanger sequencing [21]. RNA-seq, one popular NGS application, provides an efficient and cost-effective way of obtaining transcriptome data. There are a number of platforms available for generating NGS data [37,38]. Currently among the most popular are the Roche/454 FLX (http://www.roche.com) and the Illumina Hiseq 2000 (formerly Solexa; http://www.illumina.com) platforms. The Roche/454 FLX system is advantageous when longer reads are important (average read length 700 bp), whereas the Illumina system provides deeper sequencing coverage at a reduced cost per base, albeit with shorter read length (average length 100 bp).

For each platform various assemblers have emerged but during the past several years Roche 454 sequencing and the platform-specific assembler Newbler has been the most common approach for *de novo* assembly of transcriptome data [39-43]. This may be attributed to the idea that longer reads are more likely to overcome the specific challenges of *de novo* transcriptome assembly. Illumina sequencing has been used mainly when a related organism’s genome was available for reference-based assembly [44,45], although due to recently increased read length it is becoming more common for use in *de novo* assembly as well [46,47]. Several recent studies compared the performance of different sequencing platforms and assembly methods [48-50] but none of these comparisons evaluated the level of completeness or contiguity of their assemblies, nor was the performance of the assemblers evaluated without known genome information, which is the situation for any project on non-model organisms.

Our comparisons of sequencing platforms and assemblers for the Geraniaceae clearly indicated that the Illumina platform with Trinity assembly delivered the best performance in assembling a complete transcriptome in the absence of a reference genome. The Illumina assemblers (Trinity, SOAPdenovo, SOAPtrans) generated more contigs containing a greater total number of bases than the Roche/454 FLX assemblers (Newbler, MIRA). hile the MIRA assembly generated many more long contigs (>6 kb) than SOAPdenovo, the Trinity assembly out-performed all others in delivering long contigs, suggesting that the Trinity assembly contained more useful information than any of the other assemblies analyzed. While the Roche/454 FLX assemblies and the Illumina SOAPdenovo assembly produced similar results with regard to completeness and contiguity, the Illumina Trinity and SOAPtrans assemblies obtained much higher values for both parameters indicating that these assemblies comprise many more nearly complete transcripts (Figures 2 and 3).

### Functional annotation of Geraniaceae transcriptomes

A total of 58,461 (49%) and 42,506 (37%) contigs were annotated from *G. maderense* and *P.* x *hortorum*, respectively. The low percentage of annotated contigs is most likely due to the large number of total contigs assembled. The number of aligned and annotated contigs is comparable to nine other recently published transcriptomes [22,27,51-56]. The number of annotated contigs in assemblies from both Geraniaceae species was very similar for the three major categories cellular component, molecular function, and biological process (Figure 6). This is encouraging since different tissues were sampled for the two species; only one tissue, emergent leaves for *Geranium* and four tissues, emergent leaves, expanded leaves, roots and flowers for *Pelargonium*. Particularly noteworthy is the detection of genes associated with flower and embryo development and pollen-pistil interaction since flowers were not sampled for *Geranium*. Overall, this comparison indicates that there is no marked improvement in transcriptome breadth of coverage when sampling four tissues compared to only emergent leaves.

### PPR proteins and sigma factors in Geraniaceae

PPRs are a large family of RNA binding proteins encoded by over 450 genes in sequenced angiosperms. Most are organelle targeted and involved in regulating organelle gene expression [57]. Of the two classes (P and PLS) within the PPR family, those from PLS class (E and DYW subclasses) have been reported to be involved in RNA editing [14-16,58-64]. Previous studies have demonstrated correlated evolution of PLS genes and RNA editing sites in plants [17,65]. Consistent with these results, a reduction in PLS genes (Table 5) in Geraniaceae was detected, where reduced editing frequency was previously demonstrated [1]. The reduced editing frequency and reduced PPR content in Geraniaceae is especially intriguing with respect to the increased mitochondrial substitution rate in this family. Although an inverse correlation between editing frequency and substitution rate has been noted previously in Geraniaceae and other taxa [1,66-68], the finding that PPR gene content is also reduced in Geraniaceae indicates that this family is ideally suited for future studies assessing the evolutionary dynamics of editing frequency, PPR content, and mitochondrial substitution rates.

One long-standing question regarding the plastid genomes in Geraniaceae is the putative loss of the *rpoA* gene from *P.* x *hortorum*[69-71]. The complete plastid genome sequence of this species revealed several *rpoA*-like open reading frames (ORFs) that are highly divergent relative to *rpoA* genes in other angiosperms or even other Geraniaceae [11,12]. Two alternative explanations were suggested for these observations: (1) a copy of the gene in the nucleus had gained functionality; or (2) at least one of the highly divergent *rpoA*-like ORFs remains functional. Extensive evolutionary rate comparisons of plastid genes across the Geraniaceae revealed that the other three PEP subunits (*rpoB*, *rpoC1*, *rpoC2*) have significantly elevated nucleotide substitution rates and have likely experienced positive selection [8,9]. Despite exhaustive searching of the nuclear transcriptome of *P.* x *hortorum* no copy of the *rpoA* gene was detected. However, intact copies of all six sigma factors, which are required for PEP to function [72], were identified in the transcriptome. The holoparasite *Phelipanche aegyptiaca* lacks a functional PEP and mining unigene files published in a recent transcriptomic study of parasitic plants [20] failed to uncover a single sigma factor suggesting that in species where PEP sequences are lost from the plastid the requisite sigma factors are also absent from the nuclear transcriptome. The identification of all six sigma factors in the *P.* x *hortorum* transcriptome supports the likelihood that PEP is active in *P*. x *hortorum* plastids.

## Conclusions

With the widespread application of NGS techniques, the ability to process and analyze massive quantities of sequence data in a timely manner becomes imperative to a successful project. Regardless of the goals of a particular project, it is desirable to obtain data that is as accurate and complete as possible in a way that is cost effective as well as timely. In this study a cross-platform comparison of *de novo* transcriptome assembly was conducted using representative species from the two largest genera of Geraniaceae, *G. maderense* and *P.* x *hortorum*. As no reference genome is available for Geraniaceae, or any of its close relatives, this approach represents a truly *de novo* assembly allowing evaluation of efficacy among the platforms/assemblers that more closely resembles current NGS research. The assembly of Illumina Hiseq 2000 reads with Trinity or SOAPtrans was highly effective in reconstructing, as completely as is currently feasible, the protein-coding transcripts of Geraniaceae. As for the differences between the two assemblers, Trinity generated slightly more single contiguous contigs and reconstructed more reference genes with a combination of multiple contigs, while SOAPtrans ran much faster than Trinity. These differences in contiguity and completeness became more obvious with the reduced set of input data (1/40^th^ in this case). These findings recommend the Illumina platform with Trinity assembly to obtain the most complete gene coverage by a single contig, especially when a small amount of reads are available. In instances where a large amount of data is available and there are limited computational resources, Illumina SOAPtrans assembly may be preferred as it generated a relatively complete assembly much more quickly than Trinity. Furthermore, evaluation of the amount of Illumina sequence data required for generating a complete transcriptome is approximately 60 million reads.

Geraniaceae organelle genomes have been shown to exhibit a number of unusual features relative to other angiosperms, including highly accelerated rates of nucleotide substitutions in both mitochondrial and plastid genes [1,8,9], reduced RNA editing in mitochondrial genomes [1] and highly rearranged plastid genomes [10-13]. This comparative transcriptome analysis of *G. maderense* and *P.* x *hortorum* detected a reduction in PPR proteins associated with RNA editing, which corresponds with reduced RNA editing in the mitochondria. Examination of nuclear encoded, plastid targeted sigma factors required for PEP function supports the hypothesis that PEP is active in *P.* x *hortorum* plastids, possibly incorporating the product of at least one of the highly divergent *rpoA*-like ORFs in the plastid genome.

## Methods

### RNA isolation

Plant tissues were collected from live plants grown in the University of Texas (UT) greenhouse and frozen in liquid nitrogen for two species from different genera of Geraniaceae, *Geranium maderense* and *Pelargonium* x *hortorum* cv ringo white. For *Pelargonium* leaf and inflorescence samples were collected. Leaves were of two developmental stages, newly emerged and fully expanded. Entire inflorescences were harvested prior to anthesis. Root samples of *P*. x *hortorum* were harvested from specimens grown aseptically in agar media. For *Geranium*, only emergent leaves were collected. Total RNA was isolated separately from each sample type by grinding in liquid nitrogen followed by 30 min incubation at 65°C in two volumes of extraction buffer (2% Cetyltrimethylammonium bromide, 3% Polyvinylpyrrolidone-40, 3% 2-Mercaptoethanol, 25 mM Ethylenediaminetetraacetic acid, 100 mM Tris(hydroxymethyl)aminomethane-HCl pH 8, 2 M NaCl, 2.5 mM spermidine trihydrochloride) with vortexing at 5 min intervals. Phase separation with chloroform:isolamyl alcohol (24:1) was performed twice and the aqueous phase was adjusted to 2 M LiCl. Samples were precipitated overnight at 4°C and total RNA was pelleted by centrifugation at 17,000 × g for 20 min at 4°C. RNA pellets were washed once with 70% ethanol and air dried at room temperature. Following resuspension in RNase free water, RNAs were analyzed by denaturing gel electrophoresis and by spectrophotometry. For *Pelargonium*, the four tissue types were pooled in equimolar ratio. All RNAs were treated with DNase I (Fermentas, Glen Burnie MD, USA) according to the product protocol. DNase I was removed from the solution by extraction with phenol:chloroform:isoamyl alcohol (25:24:1) and the aqueous phase was adjusted to 0.3 M sodium acetate. RNA was precipitated with 2.5 volumes of cold absolute ethanol for 20 min at −80°C. Pellets were washed with 70% ethanol, air-dried and resuspended in water to 1 μg μL^-1^. Total RNA sample aliquots were frozen in liquid nitrogen and shipped on dry ice to the Beijing Genomics Institute (BGI) in Hong Kong or delivered to the Genome Sequencing Analysis Facility (GSAF) at UT. Confirmation of sample quality and concentration was conducted at each facility using the Agilent 2100 Bioanalyzer instrument (Agilent Technologies, Santa Clara CA, USA).

### Illumina sequencing

Sample preparation for Illumina sequencing was performed at BGI according to Illumina’s protocol (Part # 1004898 Rev. D). Total RNA was treated with the Ribo-Zero™ rRNA Removal Kit (Epicentre Biotechnolgies, Madison WI, USA) prior to fragmentation and priming with random hexamers for first strand cDNA synthesis using SuperScript® III Reverse Transcriptase (Invitrogen, Beijing, China). Second strand cDNA synthesis was carried out using RNase H (Invitrogen) and DNA polymerase I (New England BioLabs, Beijing, China). The resulting cDNA fragments were purified with QIAQuick® PCR extraction kit (Qiagen, Shanghai, China) and normalized with Duplex-Specific thermostable nuclease (DSN) enzyme from Kamchatka crab (Evrogen, Moscow, Russia) according to the protocol outlined by Invitrogen (Part # 15014673 Rev. C). End repair and adenylation of the normalized cDNA library was followed by ligation to the paired-end (PE) sequencing adapters. Following gel electrophoresis for size selection (180–220 bp) the library was PCR amplified for sequencing using the Illumina HiSeq™ 2000. The PE library was sequenced for 101 bp.

### Roche/454 FLX sequencing

The method for cDNA library construction and normalization was based on that of Meyer et al. [73]. Briefly, total RNA was reverse-transcribed using oligo-dT coupled to a PCR-suppression primer. The reverse complement of this primer was incorporated at the 3′ end of the first-strand cDNA using the template switching capability of the SuperScript II Reverse Transcriptase (Invitrogen). Duplex-specific nuclease was added to digest the abundant double-stranded cDNA. After purification, PCR was performed, and the product was purified and sheared by nebulization. The fragmented DNA was then end-repaired and ligated to Roche Rapid library adaptors using the NEBNext® Quick DNA Sample Prep Master Mix Set 2 and NEBNext® DNA Sample Prep Master Mix Set 2 (New England BioLabs). Final library size and concentration were measured on the Agilent BioAnalyzer and by qPCR before sequencing on the Roche/454 FLX sequencer.

### Read pre-processing

Raw reads were preprocessed to eliminate contaminant and low quality sequences. Filtering of Illumina Hiseq 2000 reads included the removal of low quality bases, reads where (poly) adenosine constitutes more than 6% of bases, and reads containing specialized features such as adaptors and other artifacts arising from library construction. Roche/454 FLX reads were preprocessed by removing reads shorter than 50 bp and reads with artificial sequences based on a vector reference file. The complete data set is available at NCBI Sequence Read Archive (Accession numbers SRA059171 for *Geranium* and SRA053016.1 for *Pelargonium*).

### Ribosomal RNA content and Illumina library complexity

Ribosomal RNA (rRNA) contigs were identified using reciprocal blast of rRNA from *Arabidopsis* (5.8S, 18S and 25S from nucleus, 5S, 16S and 23S in chloroplast, and 5S, 18S and 26S in mitochondria) as reference. The rRNA sequences from *Arabidopsis* were downloaded from TAIR [74]. Ribosomal RNA reads were removed prior to the library complexity analysis. Due to a lack of nuclear genome sequence, the remaining reads were mapped back to the whole transcriptome data using bowtie2 [75]. The mapping results were sorted using samtools [76] and then analyzed with MarkDuplicates module of Picard [32].

### Assembly

Transcritpome assemblies were initially performed on *Pelargonium* using a variety of assemblers to compare the efficacy of different platforms and assemblers. After these initial comparisons, all subsequent assembles were performed on both *Geranium* and *Pelargonium* using Trinity and Illumina data. For assembly of clean Illumina reads, Trinity [77], SOAPdenovo and SOAPtrans (http://soap.genomics.org.cn/SOAPdenovo-Trans.html) [78,79] were used. Trinity, released on 2011-08-20 (http://sourceforge.net/projects/trinityrnaseq/), was run with parameters “--seqType fq --CPU 10 --paired_fragment_length 200 --run_butterfly” on a 24-core 3.33 GHz linux work station with 1 TB memory at the Texas Advanced Computing Center (TACC, http://www.tacc.utexas.edu/). The assembly was split into three steps according to the provided script trinity.pl released with the software. The split scripts run the corresponding three steps in Trinity: inchworm, chrysalis, and butterfly. The parameters were the same for each step, and each step picked up the previous step’s output as input and processed it. The scripts will be provided by JZ upon request. The SOAPtrans assembly was run with the parameters “kmer = 61, max_rd_length = 100, avg_ins = 200” on the same server as that of Trinity. For SOAPtrans kmer lengths from 23 bp to 81 bp were explored; 61 bp was selected because it generated the best contiguity compared with other kmer values. The SOAPdenovo assembly was done at BGI on a 48-core 2.67 GHz Linux workstation with 50 GB memory with parameters “Kmer = 41, insert size = 200, overlap threshold = 50” for assembly, and “Kmer + 1” to fill the gaps. The generated fasta file was postprocessed by BGI to remove the sequences shorter than 150 bp. Assembly of Roche/454 FLX utilized MIRA [80] and Newbler [81]. MIRA 3.4.0 for a 64-bit linux system (http://sourceforge.net/projects/mira-assembler/files/MIRA/stable/) was released on 2011-08-21. MIRA was run with parameters “--job = denovo, est, accurate, 454 --fasta 454_SETTINGS” on a 12-core 3.33 GHz linux work station with 24 GB memory at TACC. Newbler 2.6 accompanies the Roche/454 FLX platform and assembly was conducted at UT GSAF on 24-core 2.40 GHz linux work station with 64 GB memory using the parameters “runAssembly -cpu 8 -urt -cdna –vt vector.fa”.

### Comparative analysis of assemblies

Trinity, SOAPdenovo and SOAPtrans assembly output comprised a single contig file each and these were used in the analyses. Unpadded fasta files were selected from the MIRA output and the isotig file was selected from the Newbler output for use in analyses.

The initial assembly quality was evaluated using the following metrics: number of assembled contigs, maximum, minimum and mean contig length, N50 and redundancy. Initial assembly statistics and contig length distribution analysis was done by custom perl scripts and MATLAB version R2011b. Contig clustering and removal of redundant contig sequences was performed using CD-HIT [82]. CD-HIT version 4.5.4 (downloaded from http://code.google.com/p/cdhit/downloads/list) was executed using parameters “cd-hit -c 1.0 -n 5 -T 12” for cDNA sequences and “cd-hit-est -c 1.0 -n 10 -T 12” for protein sequences. Redundancy was calculated from the difference between the number of contigs before and after clustering. Maximum, minimum, and mean contig length, N50 and total bases were calculated from the contigs after clustering and removal of those contigs < 200 bp.

The assemblies were aligned to two published reference databases: 357 ultra-conserved ortholog (UCO) coding sequence [35] from *Arabidopsis* (sequences available at: http://compgenomics.ucdavis.edu/compositae_reference.php), and a list of 959 single copy nuclear genes shared between *Arabidopsis*, *Oryza*, *Populus*, and *Vitis*[36] using BLASTX with evalue of 1 E-10. Contig alignment to the reference databases utilized the standalone BLAST + [83] program for 64-bit linux system (ftp://ftp.ncbi.nlm.nih.gov/blast/executables/blast+/LATEST/). The parameters for BLAST + DNA alignment were “blastn -task blastn -evalue 1 E-10 -word_size 11 -outfmt 6 -num_threads 12”. Parameters for protein alignment were “blastp -task blastp -num_threads 12 -outfmt 6”. For blastp, two different e values were used, 1 E-10 and 1 E-20, in order to address the generality of the results. Multiple sequence alignment was done by muscle [84]. Muscle for 64-bit linux system (http://www.drive5.com/muscle/downloads.htm) was used with default parameters.

The local reference database for identifying the open reading frames contained four proteomes downloaded from Phytozome (http://www.phytozome.net/search.php): *Citrus clementina*, *C. sinensis*, *Eucalyptus grandis* and *Arabidopsis thaliana*. Contigs were translated by alignment to the local database using blastx to identify open reading frames. The blastx parameter was “blastx -evalue 1e-6 -max_target_Seqs 1 -num_threads 48 –outfmt’6 std qframe”. The reading frame parameter was added to the output in order to facilitate the following analysis. The aligned regions of contigs were translated, extracted, and then extended by translating the contigs in both directions according to standard codon usage until a stop codon was encountered. The translated contigs were clustered again using CD-HIT at a threshold of 100% and all other parameters used the default settings. Two parameters, contiguity and completeness as described by Martin and Wang [85] were used to evaluate the alignment results. Briefly, contiguity is defined as the percentage of the reference transcripts covered at some arbitrary coverage threshold by a single longest contig. Completeness is defined as the percentage of the reference transcripts covered at a threshold by multiple assembled contigs (Box one in [85]). In this study a range of thresholds up to 100% was evaluated, and 80% was selected as the threshold for both contiguity and completeness calculations. Both parameters were calculated with protein sequence alignment, and the alignment results were analyzed using custom perl scripts available from JZ upon request.

### Evaluation of assemblies with different proportion of reads

To assess how much data (number of reads) is needed to construct the complete transcriptome, different proportions of sequencing data ranging from 5% to 100% were extracted for both species. The extracted reads were assembled with Trinity using the parameters described above. Extraction and assembly were repeated three times for each proportion except 100%, and the assembly statistics (contig number, contiguity and etc.) were averaged.

Basic statistics and assembly parameters such as contiguity and completeness were calculated using the same local database described above. To determine how well the assemblies cover a complete transcriptome, the custom *Arabidopsis* protein database was constructed by extracting all *Arabidopsis* proteins from Uniprot/Swissprot database [86], and protein sequences with name “hypothetical” or “predicted” were discarded. The assemblies were aligned with the database using BLASTX with an E-value of 1 E-10.

### Orthologous genes identification

Orthologous genes between transcriptomes of *G. maderense*, *P.* x *hortorum* and *A. thaliana* were identified with reciprocal blast with parameters “blastp -task blastp -num_threads 12 -max_target_Seqs 1 -evalue 1e-10 -outfmt = '6 std qlen slen”. Blast results were analyzed with custom perl scripts.

### Functional annotation

The assemblies were aligned with the NCBI nr database using BLASTX with an E-value of 1 E-6 and taking the best 10 hits for annotation. The blast results were used to annotate each sequence with gene ontology (GO) terms using Blast2GO [87-89]. To improve the efficiency of annotation, local blast2go database was downloaded (http://www.blast2go.com/b2glaunch/resources/35-localb2gdb). GO terms were mapped to the reduced GO-slim (plant) ontology to get a broader functional representation of the transcriptome.

### Identification of selected organelle targeted genes

PPR proteins were searched for using HMMER [90,91] with previously established PPR motif alignment files [92]. Transcript sequences with more than one PPR motif were considered PPR genes. Sigma factor protein sequences from *Arabidopsis* were downloaded from TAIR [74] and used as reference. Sigma factor structure and conserved domain information were obtained from previous studies [93-95]. Putative transit peptides were predicted with targetP [96,97]. Orthologs from two transcriptomes of *G. maderense* and *P.* x *hortorum* were identified by reciprocal blast at E-value 1 E-10.

## Competing interests

The authors declare that they have no competing interests.

## Authors’ contributions

JZ contributed to the design of the project, performed all analyses and drafted the manuscript; TAR isolated RNA, drafted RNA and sequencing methods sections, contributed to the design of the project, and assisted with manuscript preparation; JPM contributed to the design of the project and assisted with manuscript preparation; RKJ contributed to the design of the project and assisted with manuscript preparation. All authors read and approved the final draft of the manuscript.

## Supplementary Material

Additional file 1 **Contiguity and completeness of different protein data sets at E-value 1 E-10 (1/40**^**th **^**of the Illumina data was used by Trinity).** Click here for file

Additional file 2 **Transcriptome annotation for *Geranium maderense *** **and *Pelargonium *** **x *hortorum.***
Click here for file

## References

[B1] Parkinson CL Mower JP Qiu Y-Q Shirk AJ Song K Young ND dePamphilis CW Palmer JD Multiple major increases and decreases in mitochondrial substitution rates in the plant family Geraniaceae BMC Evol Biol 2005 13 73 10.1186/1471-2148-5-73 16368004 PMC1343592

[B2] Bakker FT Breman F Merckx V DNA sequence evolution in fast evolving mitochondrial DNA nad1 exons in Geraniaceae and Plantaginaceae Taxon 2006 13 887 896 10.2307/25065683

[B3] Cho Y Mower JP Qiu YL Palmer JD Mitochondrial substitution rates are extraordinarily elevated and variable in a genus of flowering plants Proc Natl Acad Sci U S A 2004 13 17741 17746 10.1073/pnas.0408302101 15598738 PMC539783

[B4] Mower JP Touzet P Gummow JS Delph LS Palmer JD Extensive variation in synonymous substitution rates in mitochondrial genes of seed plants BMC Evol Biol 2007 13 135 10.1186/1471-2148-7-135 17688696 PMC1973135

[B5] Sloan DB Barr CM Olson MS Keller SR Taylor DR Evolutionary rate variation at multiple level of biological organization in plant mitochondrial DNA Mol Biol Evol 2008 13 243 246 10.1093/molbev/msm266 18056075

[B6] Sloan DB Oxelman B Rautenberg A Taylor DR Phylogenetic analysis of mitochondrial substitution rate variation in the angiosperm tribe Sileneae BMC Evol Biol 2009 13 260 10.1186/1471-2148-9-260 19878576 PMC2777880

[B7] Adams KL Qiu YL Stoutemyer M Palmer JD Punctuated evolution of mitochondrial gene content: high and variable rates of mitochondrial gene loss and transfer during angiosperm evolution Proc Natl Acad Sci U S A 2002 13 9905 9912 10.1073/pnas.042694899 12119382 PMC126597

[B8] Guisinger MM Kuehl JV Boore JL Jansen RK Genome-wide analyses of Geraniaceae plastid DNA reveal unprecedented patterns of increased nucleotide substitutions Proc Natl Acad Sci U S A 2008 13 18424 18429 10.1073/pnas.0806759105 19011103 PMC2587588

[B9] Weng ML Ruhlman TA Gibby M Jansen RK Phylogeny, rate variation, and genome size evolution of *Pelargonium* (Geraniaceae) Mol Phylogen Evol 2012 13 654 670 10.1016/j.ympev.2012.05.026 22677167

[B10] Palmer JD Nugent JM Herbon LA Unusual structure of geranium chloroplast DNA: a triple-sized inverted repeat, extensive gene duplications, multiple inversions and two repeat families Proc Natl Acad Sci U S A 1987 13 769 773 10.1073/pnas.84.3.769 16593810 PMC304297

[B11] Chumley TW Palmer JD Mower JP Fourcade HM Calie PJ Boore JL Jansen RK The complete chloroplast genome sequence of *Pelargonium* x *hortorum*: organization and evolution of the largest and most highly rearranged chloroplast genome of land plants Mol Biol Evol 2006 13 2175 2190 10.1093/molbev/msl089 16916942

[B12] Guisinger MM Kuehl JV Boore JL Jansen RK Extreme reconfiguration of plastid genomes in the angiosperm family Geraniaceae: rearrangements, repeats, and codon usage Mol Biol Evol 2011 13 583 600 10.1093/molbev/msq229 20805190

[B13] Blazier CJ Guisinger MM Jansen RK Recent loss of plastid-encoded *ndh* genes within *Erodium* (Geraniaceae) Plant Mol Biol 2011 13 263 272 10.1007/s11103-011-9753-5 21327834

[B14] Kotera E Tasaka M Shikanai T A pentatricopeptide repeat protein is essential for RNA editing in chloroplasts Nature 2005 13 326 330 10.1038/nature03229 15662426

[B15] Okuda K Myouga F Motohashi R Shinozaki K Shikanai T Conserved domain structure of pentatricopeptide repeat proteins involved in chloroplast RNA editing Proc Natl Acad Sci U S A 2007 13 8178 8183 10.1073/pnas.0700865104 17483454 PMC1876591

[B16] Okuda K Chateigner-Boutin AL Nakamura T Delannoy E Sugita M Myouga F Motohashi R Shinozaki K Small I Shikanai T Pentatricopeptide repeat proteins with the DYW motif have distinct molecular functions in RNA editing and RNA cleavage in *Arabidopsis* chloroplasts Plant Cell 2009 13 146 156 10.1105/tpc.108.064667 19182104 PMC2648089

[B17] Fujii S Small I The evolution of RNA editing and pentatricopeptide repeat genes New Phytol 2011 13 37 47 10.1111/j.1469-8137.2011.03746.x 21557747

[B18] Sugiura C Kobayashi Y Aoki S Sugita C Sugita M Complete chloroplast DNA sequence of the moss *Physcomitrella patens*: evidence for the loss and relocation of *rpoA* from the chloroplast to the nucleus Nucleic Acids Res 2003 13 5324 5331 10.1093/nar/gkg726 12954768 PMC203311

[B19] Goffinet B Wickett NJ Shaw AJ Cox CJ Phylogenetic significance of the *rpoA* loss in the chloroplast genome of mosses Taxon 2005 13 353 360 10.2307/25065363

[B20] Wickett NJ Honaas LA Wafula EK Das M Huang K Wu B Landherr L Timko MP Yoder J Westwood JH dePamphilis CW Transcriptomes of the parasitic plant family Orobanchaceae reveal surprising conservation of chlorophyll synthesis Curr Biol 2011 13 2098 2104 10.1016/j.cub.2011.11.011 22169535

[B21] Kircher M Kelso J High-throughput DNA sequencing–concepts and limitations Bioessays 2010 13 524 536 10.1002/bies.200900181 20486139

[B22] Ward JA Ponnala L Weber CA Strategies for transcriptome analysis in nonmodel plants Amer J Bot 2012 13 267 276 10.3732/ajb.1100334 22301897

[B23] Pepke S Wold B Mortazavi A Computation for ChIP-seq and RNA-seq studies Nat Methods 2009 13 S22 S32 10.1038/nmeth.1371 19844228 PMC4121056

[B24] Wheat CW Rapidly developing functional genomics in ecological model systems via 454 transcriptome sequencing Genetica 2010 13 433 451 10.1007/s10709-008-9326-y 18931921

[B25] Der JP Barker MS Wickett NJ dePamphilis CW Wolf PG De novo characterization of the gametophyte transcriptome in bracken fern, *Pteridium aquilinum* BMC Genomics 2011 13 99 10.1186/1471-2164-12-99 21303537 PMC3042945

[B26] Barker MS Vogel H Schranz ME Paleopolyploidy in the Brassicales: analyses of the *Cleome* transcriptome elucidate the history of genome duplications in *Arabidopsis* and other Brassicales Genome Biol Evol 2009 13 391 399 20333207 10.1093/gbe/evp040 PMC2817432

[B27] Angeloni F Wagemaker CA Jetten MSM Camp HJMO Janssen-Megens EM Francoijs KJ Stunnenberg HG Ouborg NJ De novo transcriptome characterization and development of genomic tools for *Scabiosa columbaria* L. using next-generation sequencing techniques Mol Ecol Resour 2011 13 662 674 10.1111/j.1755-0998.2011.02990.x 21676196

[B28] Hou R Bao Z Wang S Su H Li Y Du H Hu J Wang S Hu X Transcriptome sequencing and de novo analysis for Yesso scallop (*Patinopecten yessoensis*) using 454 GS FLX PLoS One 2011 13 e21560 10.1371/journal.pone.0021560 21720557 PMC3123371

[B29] Margam VM Coates BS Bayles DO Hellmich RL Agunbiade T Seufferheld MJ Sun W Kroemer JA Ba MN Binso-Dabire CL Baoua I Ishiyaku MF Covas FG Srinivasan R Armstrong J Murdock LL Pitttendrigh BR Transcriptome sequencing, and rapid development and application of SNP markers for the legume pod borer *Maruca vitrata* (Lepidoptera: Crambidae) PLoS One 2011 13 e21388 10.1371/journal.pone.0021388 21754987 PMC3130784

[B30] Roberts SB Hauser L Seeb LW Seeb JE Development of genomic resources for Pacific Herring through targeted transcriptome pyrosequencing PLoS One 2012 13 e30908 10.1371/journal.pone.0030908 22383979 PMC3288011

[B31] Savory EA Adhikari BN Hamilton JP Vaillancourt B Buell CR Day B mRNA-Seq analysis of the Pseudoperonospora cubensis transcriptome during cucumber (Cucumis sativus L.) infection PLoS One 2012 13 e35796 10.1371/journal.pone.0035796 22545137 PMC3335787

[B32] Picard pipeline http://picard.sourceforge.net/

[B33] Tariq MA Kim HJ Jejelowo O Pourmand N Whole-transcriptome RNAseq analysis from minute amount of total RNA Nucleic Acids Res 2011 13 18 e120 10.1093/nar/gkr547 21737426 PMC3185437

[B34] Levin JZ Yassour M Adiconis X Nusbaum C Thompson DA Friedman N Gnirke A Regev A Comprehensive comparative analysis of strand-specific RNA sequencing methods Nat Methods 2010 13 9 709 715 10.1038/nmeth.1491 20711195 PMC3005310

[B35] Kozik A M M Kozik I Van Leeuwen H Van Deynze A Michelmore R Eukaryotic ultra conserved orthologs and estimation of gene capture In EST libraries Plant and Animal Genomes Conference XVI 2008 6

[B36] Duarte JM Wall PK Edger PP Landherr LL Ma H Pires JC Leebens-Mack J dePamphilis CW Identification of shared single copy nuclear genes in *Arabidopsis*, *Populus,* Vitis and Oryza and their phylogenetic utility across various taxonomic levels BMC Evol Biol 2010 13 61 10.1186/1471-2148-10-61 20181251 PMC2848037

[B37] Harismendy O Ng PC Strausberg RL Wang X Stockwell TB Beeson KY Schork NJ Murray SS Topol EJ Levy S Frazer KA Evaluation of next generation sequencing platforms for population targeted sequencing studies Genome Biol 2009 13 R32 10.1186/gb-2009-10-3-r32 19327155 PMC2691003

[B38] Metzker ML Sequencing technologies - the next generation Nat Rev Genet 2010 13 31 46 10.1038/nrg2626 19997069

[B39] Weber AP Weber KL Carr K Wilkerson C Ohlrogge JB Sampling the *Arabidopsis* transcriptome with massively parallel pyrosequencing Plant Physiol 2007 13 32 42 10.1104/pp.107.096677 17351049 PMC1913805

[B40] Novaes E Drost DR Farmerie WG Pappas GJ Jr Grattapaglia D Sederoff RR Kirst M High-throughput gene and SNP discovery in *Eucalyptus grandis*, an uncharacterized genome BMC Genomics 2008 13 312 10.1186/1471-2164-9-312 18590545 PMC2483731

[B41] Vega-Arreguin JC Ibarra-Laclette E Jimenez-Moraila B Martinez O Vielle-Calzada JP Herrera-Estrella L Herrera-Estrella A Deep sampling of the Palomero maize transcriptome by a high throughput strategy of pyrosequencing BMC Genomics 2009 13 299 10.1186/1471-2164-10-299 19580677 PMC2714558

[B42] Wall PK Leebens-Mack J Chanderbali AS Barakat A Wolcott E Liang H Landherr L Tomsho LP Hu Y Carlson JE Ma Hong Schuster SC Soltis DE Soltis PS Altman N dePamphilis CW Comparison of next generation sequencing technologies for transcriptome characterization BMC Genomics 2009 13 347 10.1186/1471-2164-10-347 19646272 PMC2907694

[B43] Cantacessi C Campbell BE Young ND Jex AR Hall RS Presidente PJA Zawadzki JL Zhong W Aleman-Meza B Loukas A Sternberg PW Gasser RB Differences in transcription between free-living and CO2-activated third-stage larvae of *Haemonchus contortus* BMC Genomics 2010 13 266 10.1186/1471-2164-11-266 20420710 PMC2880303

[B44] Nagalakshmi U Wang Z Waern K Shou C Raha D Gerst The transcriptional landscape of the yeast genome defined by RNA sequencing Science 2008 13 1344 1349 10.1126/science.1158441 18451266 PMC2951732

[B45] Rosenkranz R Borodina T Lehrach H Himmelbauer H Characterizing the mouse ES cell transcriptome with Illumina sequencing Genomics 2008 13 187 194 10.1016/j.ygeno.2008.05.011 18602984

[B46] Birol I Jackman SD Nielsen CB Qian JQ Varhol R Stazyk G Morin RD Zhao Y Hirst M Schein JE Horsman DE Connors JM Gascoyne RD Marra MA Jones SJM De novo transcriptome assembly with ABySS Bioinformatics 2009 13 2872 2877 10.1093/bioinformatics/btp367 19528083

[B47] Wang X-W Luan J-B Li J-M Bao Y-Y Zhang C-X Liu S-S De novo characterization of a whitefly transcriptome and analysis of its gene expression during development BMC Genomics 2010 13 400 10.1186/1471-2164-11-400 20573269 PMC2898760

[B48] Kumar S Blaxter ML Comparing de novo assemblers for 454 transcriptome data BMC Genomics 2010 13 571 10.1186/1471-2164-11-571 20950480 PMC3091720

[B49] Feldmeyer B Wheat CW Krezdorn N Rotter B Pfenninger M Short read Illumina data for the de novo assembly of a non-model snail species transcriptome (*Radix balthica*, *Basommatophora*, *Pulmonata*), and a comparison of assembler performance BMC Genomics 2011 13 317 10.1186/1471-2164-12-317 21679424 PMC3128070

[B50] Brautigam A Mullick T Schliesky S Weber AP Critical assessment of assembly strategies for non-model species mRNA-Seq data and application of next-generation sequencing to the comparison of C(3) and C(4) species J Exp Bot 2011 13 3093 3102 10.1093/jxb/err029 21398430

[B51] Garg R Patel RK Jhanwar S Priya P Bhattacharjee A Yadav G Bhatia S Chattopadhyay D Tyagi AK Jain M Gene discovery and tissue-specific transcriptome analysis in chickpea with massively parallel pyrosequencing and web resource development Plant Physiol 2011 13 1661 1678 10.1104/pp.111.178616 21653784 PMC3149962

[B52] Kaur S Cogan NO Pembleton LW Shinozuka M Savin KW Materne M Forster JW Transcriptome sequencing of lentil based on second-generation technology permits large-scale unigene assembly and SSR marker discovery BMC Genomics 2011 13 265 10.1186/1471-2164-12-265 21609489 PMC3113791

[B53] Logacheva MD Kasianov AS Vinogradov DV Samigullin TH Gelfand MS Makeev VJ Penin AA De novo sequencing and characterization of floral transcriptome in two species of buckwheat (*Fagopyrum*) BMC Genomics 2011 13 30 10.1186/1471-2164-12-30 21232141 PMC3027159

[B54] Natarajan P Parani M De novo assembly and transcriptome analysis of five major tissues of Jatropha curcas L. using GS FLX titanium platform of 454 pyrosequencing BMC Genomics 2011 13 191 10.1186/1471-2164-12-191 21492485 PMC3087711

[B55] Shi CY Yang H Wei CL Yu O Zhang ZZ Jiang CJ Sun J Li YY Chen Q Xia T Deep sequencing of the *Camellia sinensis* transcriptome revealed candidate genes for major metabolic pathways of tea-specific compounds BMC Genomics 2011 13 131 10.1186/1471-2164-12-131 21356090 PMC3056800

[B56] Wenping H Yuan Z Jie S Lijun Z Zhezhi W De novo transcriptome sequencing in *Salvia miltiorrhiza* to identify genes involved in the biosynthesis of active ingredients Genomics 2011 13 272 279 10.1016/j.ygeno.2011.03.012 21473906

[B57] Schmitz-Linneweber C Small I Pentatricopeptide repeat proteins: a socket set for organelle gene expression Trends Plant Sci 2008 13 663 670 10.1016/j.tplants.2008.10.001 19004664

[B58] Chateigner-Boutin AL Ramos-Vega M Guevara-Garcia A Andres C de la Luz G-NM Cantero A Delannoy E Jimenez LF Lurin C Small I CLB19, a pentatricopeptide repeat protein required for editing of *rpoA* and *clpP* chloroplast transcripts Plant J 2008 13 590 602 10.1111/j.1365-313X.2008.03634.x 18657233

[B59] Cai W Ji D Peng L Guo J Ma J Zou M Lu C Zhang L LPA66 is required for editing *psbF* chloroplast transcripts in *Arabidopsis* Plant Physiol 2009 13 1260 1271 10.1104/pp.109.136812 19448041 PMC2705037

[B60] Hammani K Okuda K Tanz SK Chateigner-Boutin AL Shikanai T Small I A study of new *Arabidopsis* chloroplast RNA editing mutants reveals general features of editing factors and their target sites Plant Cell 2009 13 3686 3699 10.1105/tpc.109.071472 19934379 PMC2798323

[B61] Robbins JC Heller WP Hanson MR A comparative genomics approach identifies a PPR-DYW protein that is essential for C-to-U editing of the *Arabidopsis* chloroplast *accD* transcript RNA 2009 13 1142 1153 10.1261/rna.1533909 19395655 PMC2685521

[B62] Yu QB Jiang Y Chong K Yang ZN AtECB2, a pentatricopeptide repeat protein, is required for chloroplast transcript *accD* RNA editing and early chloroplast biogenesis in *Arabidopsis thaliana* Plant J 2009 13 1011 1023 10.1111/j.1365-313X.2009.03930.x 19500301

[B63] Zhou W Cheng Y Yap A Chateigner-Boutin AL Delannoy E Hammani K Small I Huang J The *Arabidopsis* gene YS1 encoding a DYW protein is required for editing of *rpoB* transcripts and the rapid development of chloroplasts during early growth Plant J 2009 13 82 96 10.1111/j.1365-313X.2008.03766.x 19054358

[B64] Tseng CC Sung TY Li YC Hsu SJ Lin CL Hsieh MH Editing of *accD* and *ndhF* chloroplast transcripts is partially affected in the *Arabidopsis* vanilla cream1 mutant Plant Mol Biol 2010 13 309 323 10.1007/s11103-010-9616-5 20143129

[B65] Hayes ML Giang K Mulligan RM Molecular evolution of pentatricopeptide repeat genes reveals truncation in species lacking an editing target and structural domains under distinct selective pressures BMC Evol Biol 2012 13 66 10.1186/1471-2148-12-66 22583633 PMC3441922

[B66] Lynch M Koskella B Schaack S Mutation pressure and the evolution of organelle genomic architecture Science 2006 13 5768 1727 1730 10.1126/science.1118884 16556832

[B67] Sloan DB MacQueen AH Alverson AJ Palmer JD Taylor DR Extensive loss of RNA editing sites in rapidly evolving Silene mitochondrial genomes: selection vs. retroprocessing as the driving force Genetics 2010 13 4 1369 1380 10.1534/genetics.110.118000 20479143 PMC2927763

[B68] Cuenca A Petersen G Seberg O Davis JI Stevenson DW Are substitution rates and RNA editing correlated? BMC Evol Biol 2010 13 349 21070620 10.1186/1471-2148-10-349 PMC2989974

[B69] Downie SR Katz-Downie DS Wolfe KH Calie PJ Palmer JD Structure and evolution of the largest chloroplast gene (ORF2280): internal plasticity and multiple gene loss during angiosperm evolution Curr Genet 1994 13 367 378 10.1007/BF00351492 8082181

[B70] Palmer JD Calie PJ dePamphilis CW Logsdon JMJ Katz-Downie DS Downie SR Palmer JD Calie PJ de Pamphilis CW Logsdon JMJ Katz-Downie DS Downie SR Baltscheffsky M, Baltscheffsky M An evolutionary genetic approach to understanding plastid gene function: lessons from photosynthetic and nonphotosynthetic plants Current research in photosynthesis 1990 Amsterdam: Kluwer Academic Publishers 475 482

[B71] Palmer JD Baldauf SL Calie PJ DePamphilis CW Clegg MT, O’Brien SJ Chloroplast gene instability and transfer to the nucleus Molecular evolution 1990 New York: Alan R. Liss, Inc 97 106

[B72] Lysenko EA Plant sigma factors and their role in plastid transcription Plant Cell Rep 2007 13 845 859 10.1007/s00299-007-0318-7 17356883

[B73] Meyer E Aglyamova GV Wang S Buchanan-Carter J Abrego D Colbourne JK Willis BL Matz MV Sequencing and de novo analysis of a coral larval transcriptome using 454 GSFlx BMC Genomics 2009 13 219 10.1186/1471-2164-10-219 19435504 PMC2689275

[B74] Lamesch P Berardini TZ Li D Swarbreck D Wilks C Sasidharan R Muller R Dreher K Alexander DL Garcia-Hernandez M The *Arabidopsis* Information Resource (TAIR): improved gene annotation and new tools Nucleic Acids Res 2012 13 Database issue D1202 D1210 22140109 10.1093/nar/gkr1090 PMC3245047

[B75] Langmead B Salzberg SL Fast gapped-read alignment with Bowtie 2 Nat Methods 2012 13 4 357 359 10.1038/nmeth.1923 22388286 PMC3322381

[B76] Li H Handsaker B Wysoker A Fennell T Ruan J Homer N Marth G Abecasis G Durbin R The sequence alignment/map format and SAMtools Bioinformatics 2009 13 16 2078 2079 10.1093/bioinformatics/btp352 19505943 PMC2723002

[B77] Grabherr MG Haas BJ Yassour M Levin JZ Thompson DA Amit I Adiconis X Fan L Raychowdhury R Zeng Q Chen Z Mauceli E Hacohen N Gnirke A Rhind N di PLM F Birren BW Nusbaum C Lindblad-Toh K Friedman N Regev A Full-length transcriptome assembly from RNA-Seq data without a reference genome Nat Biotech 2011 13 644 652 10.1038/nbt.1883 PMC3571712 21572440

[B78] Li R Li Y Kristiansen K Wang J SOAP: short oligonucleotide alignment program Bioinformatics 2008 13 713 714 10.1093/bioinformatics/btn025 18227114

[B79] Li R Zhu H Ruan J Qian W Fang X Shi Z Li Y Li S Shan G Kristiansen K Li S Yang H Wang J Wang J De novo assembly of human genomes with massively parallel short read sequencing Genome Res 2010 13 265 272 10.1101/gr.097261.109 20019144 PMC2813482

[B80] Chevreux B Pfisterer T Drescher B Driesel AJ Muller WEG Wetter T Suhai S Using the miraEST assembler for reliable and automated mRNA transcript assembly and SNP detection in sequenced ESTs Genome Res 2004 13 1147 1159 10.1101/gr.1917404 15140833 PMC419793

[B81] Margulies M Egholm M Altman WE Attiya S Bader JS Bemben LA Berka J Braverman MS Chen YJ Chen Z Dewell SB Du L Fierro JM Gomes XV Godwin BC He W Helgesen S Ho CH Irzyk GP Jando SC Alenquer MLI Jarvie TP Jirage KB Mim J-B Knight JR Lanza JR Leamon JH Lefkowitz SM Genome sequencing in microfabricated high-density picolitre reactors Nature 2005 13 376380 10.1038/nature03959 PMC1464427 16056220

[B82] Li W Godzik A CD-HIT: a fast program for clustering and comparing large sets of protein or nucleotide sequences Bioinformatics 2006 13 1658 1659 10.1093/bioinformatics/btl158 16731699

[B83] Camacho C Coulouris G Avagyan V Ma N Papadopoulos J Bealer K Madden TL BLAST+: architecture and applications BMC Bioinforma 2009 13 421 10.1186/1471-2105-10-421 PMC2803857 20003500

[B84] Edgar RC MUSCLE: multiple sequence alignment with high accuracy and high throughput Nucleic Acids Res 2004 13 1792 1797 10.1093/nar/gkh340 15034147 PMC390337

[B85] Martin JA Wang Z Next-generation transcriptome assembly Nat Rev Genet 2011 13 671 682 10.1038/nrg3068 21897427

[B86] Boeckmann B Bairoch A Apweiler R Blatter MC Estreicher A Gasteiger E Martin MJ Michoud K O'Donovan C Phan I Pilbout S Schneider M The SWISS-PROT protein knowledgebase and its supplement TrEMBL in 2003 Nucleic Acids Res 2003 13 365 370 10.1093/nar/gkg095 12520024 PMC165542

[B87] Conesa A Gotz S Blast2GO: a comprehensive suite for functional analysis in plant genomics Int J Plant Genomics 2008 13 619832 18483572 10.1155/2008/619832 PMC2375974

[B88] Conesa A Gotz S Garcia-Gomez JM Terol J Talon M Robles M Blast2GO: a universal tool for annotation, visualization and analysis in functional genomics research Bioinformatics 2005 13 3674 3676 10.1093/bioinformatics/bti610 16081474

[B89] Gotz S Garcia-Gomez JM Terol J Williams TD Nagaraj SH Nueda MJ Robles M Talon M Dopazo J Conesa A High-throughput functional annotation and data mining with the Blast2GO suite Nucleic Acids Res 2008 13 3420 3435 10.1093/nar/gkn176 18445632 PMC2425479

[B90] Eddy SR Profile hidden Markov models Bioinformatics 1998 13 755 763 10.1093/bioinformatics/14.9.755 9918945

[B91] Lurin C Andres C Aubourg S Bellaoui M Bitton F Bruyere C Caboche M Debast C Gualberto J Hoffmann B Lecharnt A Ret ML Martin-Magniette M-L Mireau H Peeters N Renou J-P Szurek B Taconnat L Small I Genome-wide analysis of *Arabidopsis* pentatricopeptide repeat proteins reveals their essential role in organelle biogenesis Plant Cell 2004 13 2089 2103 10.1105/tpc.104.022236 15269332 PMC519200

[B92] Small ID Peeters N The PPR motif - a TPR-related motif prevalent in plant organellar proteins Trends Biochem Sci 2000 13 46 47 10664580 10.1016/s0968-0004(99)01520-0

[B93] Helmann JD Chamberlin MJ Structure and function of bacterial sigma factors Annu Rev Biochem 1988 13 839 872 10.1146/annurev.bi.57.070188.004203 3052291

[B94] Isono K Shimizu M Yoshimoto K Niwa Y Satoh K Yokota A Kobayashi H Leaf-specifically expressed genes for polypeptides destined for chloroplasts with domains of sigma70 factors of bacterial RNA polymerases in *Arabidopsis thaliana* Proc Natl Acad Sci U S A 1997 13 14948 14953 10.1073/pnas.94.26.14948 9405719 PMC25143

[B95] Hakimi MA Privat I Valay J-G Lerbs-Mache S Evolutionary conservation of C-terminal domains of primary sigma(70)-type transcription factors between plants and bacteria J Biol Chem 2000 13 9215 9221 10.1074/jbc.275.13.9215 10734058

[B96] Nielsen H Engelbrecht J Brunak S Heline G Identification of prokaryotic and eukaryotic signal peptides and prediction of their cleavage sites Protein Eng 1997 13 1 6 10.1093/protein/10.1.1 9051728

[B97] Emanuelsson O Nielsen H Brunak S Heline G Predicting subcellular localization of proteins based on their N-terminal amino acid sequence J Mol Biol 2000 13 1005 1016 10.1006/jmbi.2000.3903 10891285

