# Supplementary figures and images for: Comparative analyses of two Geraniaceae transcriptomes using next-generation sequencing
Source: BMC Plant Biol. 2013 Dec 29;13:228. doi: 10.1186/1471-2229-13-228 (PMC3880972; doi:10.1186/1471-2229-13-228)

**A**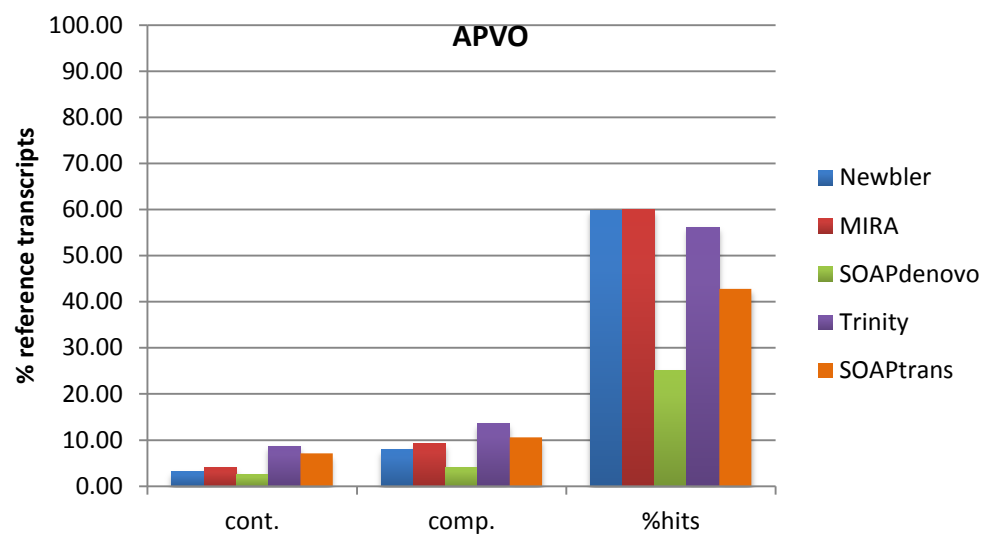**B**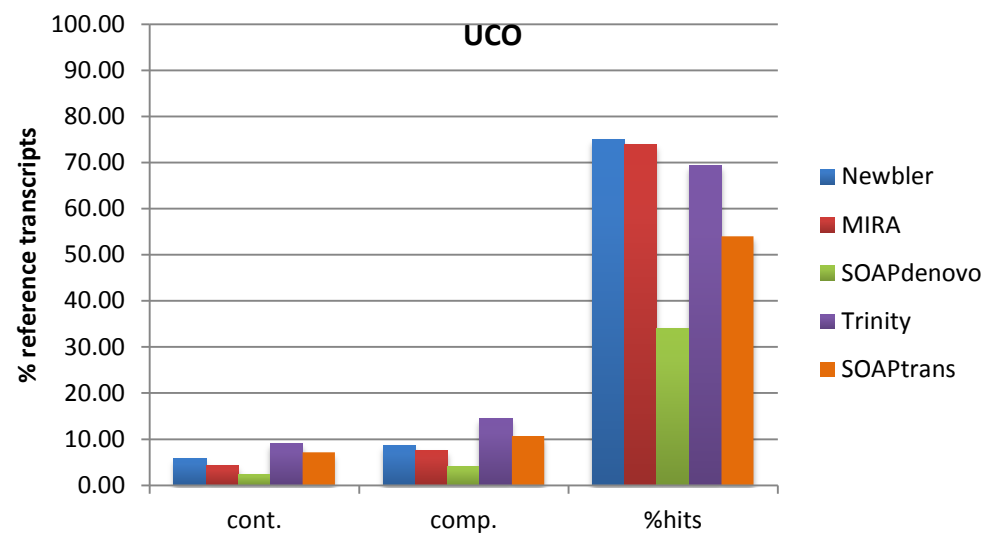

Supplement: Additional file 1 — Contiguity and completeness of different protein data sets at E-value 1 E-10 (1/40 th of the Illumina data was used by Trinity). [file 1471-2229-13-228-S1.pdf]
